# Fecal Microbiome Characteristics and the Resistome Associated With Acquisition of Multidrug-Resistant Organisms Among Elderly Subjects

**DOI:** 10.3389/fmicb.2019.02260

**Published:** 2019-09-27

**Authors:** Rafael Araos, Thomas Battaglia, Juan A. Ugalde, Marcelo Rojas-Herrera, Martin J. Blaser, Erika M. C. D’Agata

**Affiliations:** ^1^Instituto de Ciencias e Innovación en Medicina, and Millennium Initiative for Collaborative Research on Bacterial Resistance (MICROB-R), Facultad de Medicina Clínica Alemana, Universidad del Desarrollo, Santiago, Chile; ^2^Centre for Integrative Bioinformatics (IBIVU), VU University of Amsterdam, Amsterdam, Netherlands; ^3^Center for Advanced Biotechnology and Medicine, Rutgers University, New Brunswick, NJ, United States; ^4^Rhode Island Hospital, Brown University, Providence, RI, United States

**Keywords:** antibiotic resistance, geriatrics, multidrug-resistant organisms, whole metagenome shotgun sequencing, 16S rRNA amplicon sequencing

## Abstract

Infections caused by multidrug-resistant organisms (MDRO) lead to considerable morbidity and mortality. The elderly population residing in nursing homes are a major reservoir of MDRO. Our objective was to characterize the fecal microbiome of 82 elderly subjects from 23 nursing homes and compare their resistome to that of healthy young persons. Comparisons of microbiome composition and the resistome between subjects who acquired MDRO or not were analyzed to characterize specific microbiome disruption indices (MDI) associated with MDRO acquisition. An approach based on both 16S rRNA amplicon and whole metagenome shotgun (WMS) sequencing data was used. The microbiome of the study cohort was substantially perturbed, with *Bacteroides*, Firmicutes, and Proteobacteria predominating. Compared to healthy persons, the cohort of elderly persons had an increased number, abundance, and diversity of antimicrobial resistance genes. High proportions of study subjects harbored genes for multidrug-efflux pumps (96%) and linezolid resistance (52%). Among the 302 antimicrobial resistance gene families identified in any subject, 60% were exclusively detected within the study cohort, including Class D beta-lactamase genes. Subjects who acquired MDRO or not had significant differences in bacterial taxa; *Odoribacter laneus*, and *Akkermansia muciniphila* were significantly greater among subjects who did not acquire MDRO whereas *Blautia hydrogenotrophica* predominated among subjects who acquired MDRO. These findings suggest that specific MDI may identify persons at high risk of acquiring MDRO.

## Introduction

Rates of infections caused by multidrug-resistant organisms (MDRO), including methicillin-resistant *Staphylococcus aureus* (MRSA), vancomycin-resistant enterococci (VRE) and multidrug-resistant gram-negative bacteria (MDRGN) continue to increase worldwide (Diekema et al., 2019). In the United States, data from 2008–2011 showed that over 2 million infections were caused by MDRO annually with 23,000 attributable deaths, which is considered a critical public health threat (Marston et al., 2016). Current preventive efforts to curtail MDRO infections in the hospital setting focus on preventing cross-transmission and acquisition by healthcare workers by donning gowns and gloves and improving hand hygiene compliance (Siegel et al., 2007). Since exposure to antimicrobials is among the greatest risk factor for the emergence and spread of MDRO (Joint Commission on Hospital Accreditation, 2016), decreasing unnecessary antimicrobial use is a major focus. Despite such interventions, MDRO rates continue to rise, and therefore novel approaches are needed.

There is a strong association between imbalances of the intestinal microbiome, also referred as dysbiosis, and increased risk of MDRO colonization and infections (Pamer, 2016; Iacob et al., 2019). Intestinal domination by *Enterococcus* spp. and Proteobacteria, defined as >30% of the microbiome, increased the risk of vancomycin-resistant enterococcal bacteremia by 9-fold and gram-negative rod bacteremia by 5-fold, respectively, (Taur et al., 2012). Conversely, particular taxa, such as *Lactobacillus* spp. have been associated with protective effects against MDRO colonization (Araos et al., 2016). Since manipulating the gut microbiome presents a potential target for infection prevention, greater understanding of its structure and composition can identify subjects at high risk of MDRO colonization or infection. For example, a recent randomized double-blinded, placebo-controlled trial of a microbiota-based drug, RBX2660, was promising in the prevention of recurrent *Clostridioides difficile* infection (Dubberke et al., 2018).

Rates of MDRO colonization and infection are among the highest in elderly persons residing in nursing homes (Mody et al., 2018). In a 5-year study of MDRO colonization and acquisition rates among 362 residents with advanced dementia, 46% were colonized with at least one MDRGN at enrollment, and 36% acquired at least one MDRGN over the 12-month study period (Mitchell et al., 2014). A better understanding of the overall fecal microbiome among the elderly with advanced dementia and developing specific microbiome disruption indices (MDI) that can identify subjects at high risk of MDRO acquisition are needed. Since this patient population is a major reservoir of MDRO, investigating their fecal resistomes to characterize and quantify fecal antimicrobial-resistance (AR) genes is needed (van Schaik, 2015). As such, the goals of this study were to characterize the fecal microbiome and resistomes in a cohort of elderly subjects with advanced dementia and to identify the MDI associated with MDRO acquisition.

## Materials and Methods

In this metagenomic study of subjects with advanced dementia, four analyses were performed. First, the structure and composition of the fecal microbiome were characterized within the entire cohort. Second, the diversity and abundance of predicted AR genes in the subjects’ fecal resistomes were analyzed and compared to the resistomes of 82 healthy persons reported by Kaminski et al. (2015) whose fecal samples were sequenced using a similar approach (Human Microbiome Project Consortium, 2012). Third, to identify unique characteristics of the fecal microbiome that may predict MDRO acquisition, the microbiome composition between residents who acquired MDRO or not, was compared. Lastly, the resistomes of study subjects were compared between those who acquired MDRO or not. The taxonomical analyses of the fecal microbiomes were performed using both 16S rRNA amplicon and whole metagenome shotgun (WMS) sequencing data and results were compared. The institutional review board at Rhode Island Hospital, Providence, RI, United States approved this study.

### Patient Population and Sample Collection

Patient data and rectal samples were obtained from The Study of Pathogen and Antimicrobial Use in Dementia (SPREAD), a prospective cohort of 362 residents with advanced dementia in 35 nursing homes from the Boston, Massachusetts area, which quantified antimicrobial prescribing patterns for suspected infections and the acquisition of MDRO (Mitchell et al., 2014). In this cohort study, data and rectal samples were collected at baseline and every 3 months for up to 12 months or until subject death, and included demographics, cause of dementia, comorbidities, functional status, and antimicrobial exposures. Inclusion criteria for this study required that subjects from the SPREAD study had two or more consecutive rectal samples and were exposed to systemic antimicrobials. MDRO acquisition was defined as a negative baseline swab at recruitment and at least one positive subsequent swab for MDRO. The follow-up time was defined as the number of days from the baseline swab to the first swab positive for an MDRO. A cross-sectional characterization of MDI associated with MDRO acquisition, was performed using the rectal swab collected preceding detection of MDRO acquisition. For subjects who did not acquire MDRO, the last available rectal swab during the study period was analyzed.

From each subject, sterile double tipped swabs (Starswab II; Starplex Scientific Inc., Ontario, Canada) were inserted into the anus. The first swab was used to identify MDRO using culture-dependent techniques as previously described (Snyder and D’Agata, 2012; Araos et al., 2016), and the second was frozen at −80°C in 20% glycerol for further assessments of microbial communities. MDRO identified by culture included VRE, MRSA, and MDRGN. MDRGN were defined as gram-negative organisms resistant to ≥3 of the following: ampicillin/sulbactam, or piperacillin/tazobactam, ceftriaxone or ceftazidime (ceftazidime only for *Pseudomonas aeruginosa*), ciprofloxacin, gentamicin, or meropenem (Lake et al., 2018). Species identification and susceptibility testing were performed per Clinical and Laboratory Standards Institute methods (Em 100 Connect - Clsi M100 ED29, 2019).

Patient’s characteristics were reported using descriptive statistics. Categorical variables were described using absolute frequencies with percentages, and continuous variables were described using mean and standard deviation (SD) or median and interquartile range (IQR) according to the distribution of the variables. Comparisons of clinical and epidemiological variables between subjects that acquired MDRO or not were performed using Fisher exact test or Wilcoxon rank–sum test for categorical variables and continuous variables, respectively. A significance level of *P* < 0.05 was used for all statistical tests. All statistical analyses performed for the general characterization of the study population were performed using Stata software v. 13.0.

### Assessment of the Fecal Microbiomes and Resistomes

Frozen rectal swabs retrieved from the SPREAD study sample repository were thawed and immediately placed into 96-well microtiter plate. Fecal DNA was extracted using the PowerSoil DNA Isolation Kit (MOBIO, West Carlsbad, CA) according to the manufacturer’s protocol. The concentration of extracted DNA was determined by Nanodrop 1000 (Thermo Scientific, Waltham, MA, United States), and DNA was stored at −20°C until used. Subsequently, samples underwent 16S rRNA amplicon sequencing and WMS, as described below.

#### 16S rRNA Amplicon Sequencing and Analyses

All samples were amplified and barcoded for multiplex pyrosequencing using primers targeted to the V4 region of the bacterial 16S rRNA gene under uniform polymerase chain reaction (PCR) conditions that included 3 min at 94°C and 45 cycles of 45 s at 94°C, 60 s at 50°C, and 90 s at 72°C with final extension for 10 min at 72°C (Caporaso et al., 2010). We used forward primer (AAT GAT ACG GCG ACC ACC GAG ATC TAC ACT ATG GTA ATT GTG TGC CAG CMG CCG CGG TAA) that includes a 5′ Illumina adaptor, forward primer pad, 2 bp linker, and the 515F 16S rRNA primer, and reverse primer (CAA GCA GAA GAC GGC ATA CGA GAT NNNNNNNNNNNN –AGT CAG TCA G-CC-GGA CTA CHV GGG TWT CTA AT) that includes the Illumina 3′ adapter with a 12-nt error-correcting Golay barcode, reverse primer pad, 2 bp linker and the 806R 16S rRNA primer. PCRs were run in triplicate using 0.2 μM of the primers, 1 μl of template and 1X HotMasterMix (5 PRIME, Gaithersburg, MD, United States), and the products were cleaned using a PCR Purification Kit (Qiagen) after pooling. Cleaned PCR products were quantified using the Qubit dsDNA HS Assay Kit (Invitrogen^TM^, Eugene OR), then adjusted to optimal molarity, as described (Iizumi et al., 2016). Sequencing was performed using the Illumina MiSeq platform in the New York University Langone Medical Center (NYULMC) Genome Technology Core.

For the overall assessment of the structure and composition of the fecal microbiomes and the comparison of microbiomes between subjects who acquired MDRO or not, demultiplexed sequences were curated and analyzed using the DADA2 pipeline (Callahan et al., 2016). All sequences were filtered to truncate the paired reads to 150 nt and eliminate reads with quality values <2. Error rates were estimated and corrected by pooling all the reads from the sequencing run, with default parameters. The one sample that yielded less than 1,500 reads was excluded from downstream analyses. Taxonomy was assigned using the SILVA 123 database. Resulting amplicon sequence variants (ASVs) were analyzed using the Phyloseq package in R (McMurdie and Holmes, 2013). Within-sample diversity (alpha diversity) was estimated by calculating the Shannon diversity index (evenness) and the total number of observed ASV (richness) and then compared between subjects that acquired MDRO or not using non-parametric testing (Wilcoxon rank–sum test). Correlations between alpha diversity and relevant subject characteristics such as age, duration of follow-up and antimicrobial exposure were also analyzed based on the Spearman coefficient. Between-sample diversity (beta diversity) was determined by estimating UniFrac distances between samples. Between sample distances were visualized using principal coordinates analysis and sample-clustering patterns were investigated using the ADONIS test according to the metadata of interest. Differentially abundant features between subjects that acquired MDRO or not were compared using DESeq2, setting an alpha value ≤0.05 and correcting for multiple comparisons using the Benjamini-Hochberg method with a false discovery rate of 5%.

#### Whole Metagenome Shotgun Sequencing and Analyses

Fecal samples were used for WMS sequencing, with 150-bp paired-end reads (2 × 150), across 1–2 lanes of an Illumina HiSeq 4000 platform. Quality filtering was performed by removing host-contaminated sequences using KneadData and applying conservative quality threshold criteria of Phred score >20 and minimum sequence length >105-bp, using Cutadapt (Martin, 2011). Following quality filtering, the marker-based tool, MetaPhlan2 (v.2.6.0) was used to profile the composition of microbial communities, which utilized a database of ∼1 million clade-specific markers from >17,000 reference genomes (Truong et al., 2015). The biomarker identification tool, LEfSe (Linear discriminant analysis Effect Size) was used to identify differentially abundant features between subjects that acquired MDRO or not, implemented by a non-parametric factorial Kruskal–Wallis sum–rank test using default parameters (alpha level of 0.05 and LDA score ≥2) (Segata et al., 2011).

To quantify antibiotic resistance protein sequences in the metagenomic data, high-quality sequences were aligned against a curated resistome database using the protein marker alignment tool, ShortBRED (Short, Better Representative Extract Dataset), with the 151 bp antibiotic resistance markers used as the alignment reference (Kaminski et al., 2015) and default parameters of >95% sequence identity and 95% of the query sequence length. Sample abundance tables were normalized by default to Reads Per Kilobase of transcript per Million mapped reads (RPKM) to account for differences in gene length and sampling depth. Figures were generated using R-programing language and R-library ggplot2 (Wickham, 2009) and Prism 8. The abundance and diversity of AR genes in the study population were compared to the HMP cohort by using AR gene data directly from the ShortBRED publication to re-calculate descriptive statistics (Kaminski et al., 2015). Finally, differentially abundant AR genes between subjects that acquired MDRO or not were investigated using LEfSe with default parameters (Segata et al., 2011).

## Results

### Characteristics of the Study Population

Among 362 subjects in the SPREAD study, 87 subjects, from 23 different nursing homes, met the inclusion criteria. Fecal samples from five subjects could not be successfully sequenced, resulting in a cohort of 82 subjects. Demographics, clinical characteristics, type of dementia and details on antimicrobial exposure are shown in Table 1.

**TABLE 1:** Characteristics and antimicrobial exposure of the study population of 82 elderly persons in nursing homes.

**Patient characteristics^∗^**	**Study population**
Mean age ± SD	86.5 ± 7.6
Male	10 (12.2)
Caucasian	74 (90.2)
Alzheimer’s disease	64 (78.1)
Diabetes mellitus	20 (24.4)
Chronic obstructive pulmonary disease	9 (11)
Chronic heart failure	13 (15.9)
**Antimicrobial exposure prior to rectal sampling**	
Quinolones	25 (30.5)
Extended-spectrum cephalosporins	13 (15.9)
Trimethoprim/sulfamethoxazole	13 (15.9)
Cefazolin	8 (9.8)
Nitrofurantoin	7 (8.5)
Piperacillin/tazobactam	6 (7.3)
Penicillins	5 (6.1)
Azithromycin	4 (4.9)
Vancomycin	4 (4.9)
Doxycycline	2 (2.4)
Carbapenems	1 (1.2)
Gentamicin	1 (1.2)
Linezolid	1 (1.2)

*^∗^Absolute number (%) unless specified. Abbreviations: SD, standard deviation; Quinolones, ciprofloxacin or levofloxacin; Extended-spectrum cephalosporins, ceftriaxone or ceftazidime; Penicillins, ampicillin or penicillin; Carbapenems, meropenem or imipenem.*

### Microbial Diversity and Composition of the Fecal Microbiomes of the Study Population Using 16S rRNA Amplicon Sequencing Data

Eighty-two samples underwent 16S rRNA amplicon sequencing and yielded 624,291 high-quality sequence reads, with a median (IQR) of 7,816 (6,830–8,885) reads per sample. One sample was excluded from downstream analysis as it provided less than 1,500 reads. A total of 1,345 ASVs present in at least one sample were inferred from the data set. The median microbial diversity (IQR) estimated using the Shannon index (evenness) and the observed number of ASVs (richness) among the study population was 3.27 (2.67–3.7) and 168 (116–213), respectively. There was no significant correlation between the Shannon index and subject age, duration of follow-up, or the number of antimicrobial courses before fecal sample collection (*P* > 0.05). In total, 13 phyla, 70 families, and 245 genera were detected (Table 2), with a predominance of taxa associated with human infections. Notably, Proteobacteria was the second most abundant phylum and *Enterobacteriaceae* was the most abundant family in the fecal microbiome of the study cohort.

**TABLE 2:** Comparison of relative abundances of the most abundant (>1%) taxa at the Phylum and family level and most represented taxa at the Genus level (>75% of samples at >1% abundance) by 16S rRNA and whole metagenome shotgun (WMS) sequencing for the group of 82 subjects.

	**Relative abundance (%)**	
	
	**16S rRNA amplicon sequencing**	**WMS sequencing**
**Phylum**		
Firmicutes	40.9	29.3
Proteobacteria	24.9	27.1
Bacteroidetes	21.5	25.9
Actinobacteria	7.8	10.8
Verrucomicrobia	3.4	1.1
**Family**		
*Enterobacteriaceae*	16.8	16.7
*Clostridiales Family_XI*	11.7	4.5
*Bacteroidaceae*	11.4	10.8
*Ruminococcaceae*	9.2	8.4
*Lachnospiraceae*	6.4	3.0
*Porphyromonadaceae*	6.2	9.8
*Campylobacteraceae*	6.0	7.7
*Corynebacteriaceae*	4.0	3.0
*Staphylococcaceae*	3.4	1.1
*Verrucomicrobiaceae*	3.4	1.1
*Prevotellaceae*	2.6	2.4
*Aerococcaceae*	2.2	1.5
*Bifidobacteriaceae*	2.0	4.9
*Enterococcaceae*	1.8	2.2
*Rikenellaceae*	1.5	2.8
*Actinomycetaceae*	1.4	1.3
*Lactobacillaceae*	1.1	1.2
*Clostridiaceae*	<1	2.8
**Genus**		
*Bacteroides* spp.	11.9	10.8
*Escherichia* spp.	7.9	7.8
*Campylobacter* spp.	6.2	7.7
*Proteus* spp.	7.2	6.8
*Peptoniphilus* spp.	3.7	1.3
*Corynebacterium* spp.	3.7	3.0
*Staphylococcus* spp.	3.3	1.1
*Porphyromonas* spp.	2.7	5.1
*Finegoldia* spp.	2.6	2.1
*Bifidobacterium* spp.	2.0	4.7
*Enterococcus* spp.	1.8	2.1
*Lachnoclostridium* spp.	1.8	ND
*Alistipes* spp.	1.5	2.8
*Clostridium* spp.	<1	2.7

*ND, not detected; WMS, whole metagenome shotgun sequencing.*

### Microbial Diversity and Composition of the Fecal Microbiomes of the Study Population Using WMS Sequencing Data

Whole metagenome shotgun sequencing of the decontaminated and quality-filtered set of the 82 rectal swabs (Four samples sequenced on a single lane, and 78 on two lanes), yielded average sample depth of 6.1 Gbp (±3.8), and read number of 4.0E + 07 ± (2.5E + 07). Host reads accounted for 27.6% (±31.7) ranging from 0.2 to 95.8%. Importantly, the samples that had high (>80%) levels of non-microbial DNA retained at least ∼5 million high quality reads to be used for analyses. Therefore, all samples were included in downstream analyses.

Similar to the findings of the 16S rRNA gene-targeted sequencing, overall within-sample microbial diversity was low. The median (IQR) Shannon index and the number of observed ASVs were 2.77 (2.18–3.1) and 102 (83–118), respectively. The fecal microbiome of the study population was dominated by Bacteria, representing 95.1% of the microbial community, followed by viral sequences (4.3%), Archaea (0.6%) and Fungi (0.02%). Taxa at the Phylum and Family level at mean relative abundance >1% and the more prevalent Genera (≥75% of samples with a mean relative abundance >1%) are shown in Table 2. Again, a predominance of Proteobacteria and microbiota belonging to the *Enterobacteriaceae* family was observed.

### Abundance and Diversity of AR Genes in the Fecal Microbiomes of the Study Cohort

**FIGURE 1:**
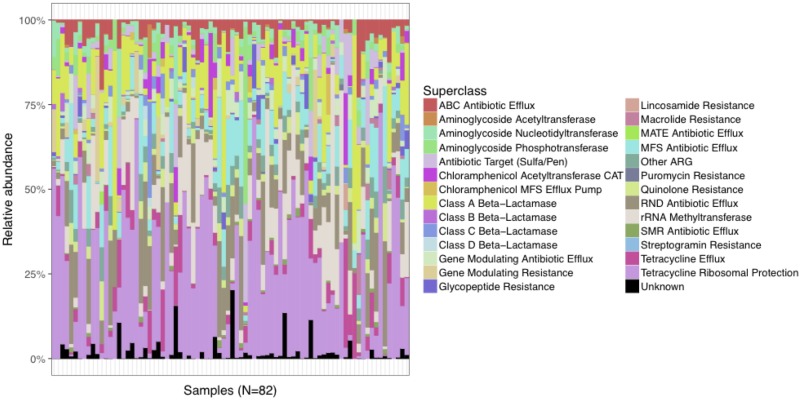
Distribution of relative abundances of the most abundant AR gene superclasses among the 82 subjects in the study cohort. Every subject had one or more tetracycline-resistance gene, and >90% had at least one AR gene classified as Class A Beta-Lactamase, RND Antibiotic Efflux, MFS Antibiotic Efflux, Aminoglycoside Phosphotransferase, Mutational Quinolone Resistance, rRNA Methyltransferase, ABC Antibiotic Efflux, and Gene Modulating Antibiotic Efflux.

The relative abundance of predicted AR genes in the fecal microbiome of the study population normalized to the total number of reads was 114,740 RPKM that grouped into 295 families. The majority of AR genes were observed at very low relative abundances, with only 50 AR gene families present in relative abundances >1%. The 295 AR gene families were clustered into more inclusive superclasses according to their hypothetical phenotype. The content of AR gene superclasses was highly heterogeneous. The most abundant AR gene superclasses (overall total number of RPKM) were tetracycline ribosomal protection (16,883), RND antibiotic efflux (15,183), MFS antibiotic efflux (12,272), aminoglycoside phosphotransferase (8,564), and class A beta-lactamase (8,416) (Figure 1). These highly abundant AR gene superclasses were highly prevalent in the study population: every subject had at least one tetracycline resistance determinant, 98.8% had at least one AR gene classified as class A beta-lactamase, 98.8% as RND antibiotic efflux, 96.3% as MFS antibiotic efflux, and 92.7% as aminoglycoside phosphotransferase. Other AR gene superclasses present in >90% of the samples included mutational quinolone resistance, rRNA methyltransferase, ABC antibiotic efflux, and gene modulating antibiotic efflux. A comprehensive list of the AR genes found in the fecal microbiome of the study participants, grouped by family, class, and superclass, is shown in Supplementary Table S2.

The total abundance of AR genes (RPKM) was not significantly correlated with the observed taxonomical diversity. Beta diversity was assessed by estimating the Bray-Curtis dissimilarity index between samples according to their AR gene content. The ordination analysis did not show any specific clustering pattern according to the clinical metadata examined, which included the presence of dementia, congestive heart failure, chronic obstructive pulmonary disease, diabetes mellitus, as well as the nursing home of origin. In total, these data indicated that the fecal microbiome of the study population carries a large and diverse resistome.

### Comparison of the Fecal Resistomes of the Study Population to Those From a Healthy Population Unexposed to Antimicrobials

**FIGURE 2:**
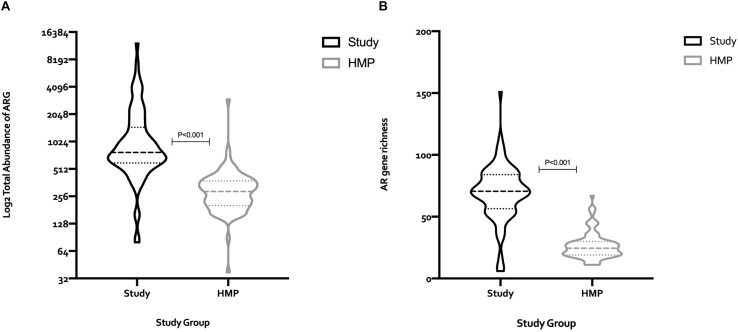
Comparison of the total number of AR genes (RPKM), and AR gene richness between the 82 subjects in the study cohort, and 82 subjects from the human microbiome project (HMP). The median (IQR) number of AR genes among study subjects and HMP participants was 776.8 (594.6–1469) and 289.1 (202.1–378.4), respectively, **(A)**. The median (IQR) AR gene richness among subjects in the study cohort and the HMP was 70.5 (56.5–84) vs. 24.5 (19–30), respectively, **(B)**. Statistical differences between groups were determined using the Mann–Whitney test. RPKM, reads per kilobase of transcript per million mapped reads; HMP, human microbiome project; ARG, antimicrobial resistance genes.

**FIGURE 3:**
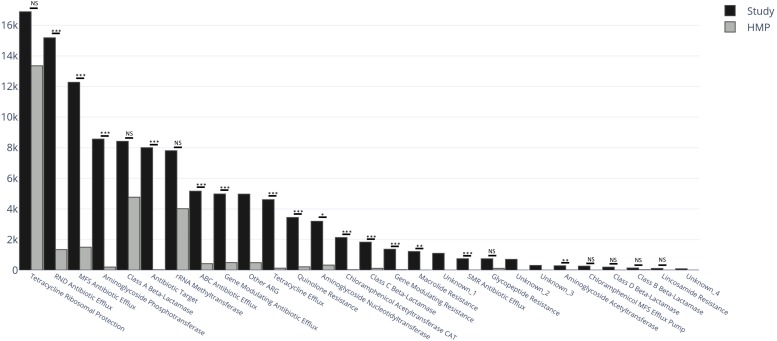
Total abundance of AR genes (RPKM) within AR gene superclasses among the 82 subjects in the study cohort (black) and 82 healthy subjects from the human microbiome project (HMP) (gray). Between-group differences in the distribution of each AR gene superclass were estimated by comparing medians using Kruskal–Wallis Rank Sum test with subsequent Dunn’s correction for multiple comparisons. The categories Unknown_1, _2, _3 and _4, and Other ARG, were not considered in the analyses. RPKM, reads per kilobase of transcript per million mapped reads; ARG, antimicrobial resistance genes; NS, non-significant (*P* > 0.05); ^∗^*P* = 0.03 ^∗∗^*P* = 0.002 ^∗∗∗^*P* < 0.001.

To assess whether the fecal microbiomes of this high-risk population were skewed in relation to healthy adults, we compared findings with those from the Human Microbiome Project (HMP). AR gene abundance in the study cohort was four-fold higher than in the HMP cohort (114,740 RPKM vs. 27,621 RPKM, respectively) (Figure 2A), and the diversity of the AR genes estimated by their within-sample richness was significantly higher as well (Figure 2B). Figure 3 shows the differences in the total abundance of AR gene superclasses between study subjects and those from the HMP. These results provide evidence that factors such as age and antimicrobial exposure may be crucial modifiers of the human fecal resistome.

The study cohort contained AR genes from more families than the HMP cohort (295 vs. 120 families) (full listing in Supplementary Table S1). Among the 302 AR gene families identified between the two groups, 182 (60.3%) were found exclusively within the study cohort, 7 (2.3%) were found exclusively in the HMP cohort and 113 (37.4%) in both. Five superclasses were unique to the study cohort: class D beta-lactamase (19.5% of subjects), chloramphenicol MFS efflux pump (19.5%), MATE antibiotic efflux (4.9%), puromycin resistance (3.7%), and streptogramin resistance (1.2%); there were no superclasses unique to the HMP cohort. The *qnr* gene, a plasmid-mediated quinolone resistance determinant, was only detected in the study cohort [in 4 (4.9%) subjects] at an abundance of 425 RPKM (Kehrenberg et al., 2007). *cfr*, a gene that encodes for a 23S rRNA methyltransferase and has the potential to confer resistance to linezolid, was present in 43 (52.4%) study subjects at an abundance of 45 RPKM and in 7 (8.5%) in the HMP cohort at an abundance of 0.6 RPKM.

### Analysis of Clinical Characteristics and the Fecal Microbiome of Subjects That Acquired MDRO or Not Using 16S rRNA and WMS Sequencing

Among the 82 subjects, 29 (35.4%) acquired a total of 32 MDRO isolates. These included MRSA (Five subjects), VRE (1 patient) and 26 multidrug-resistant gram-negative isolates: *Escherichia coli* (10 subjects), *Proteus mirabili*s (5), *P. aeruginosa* (3), *Morganella morganii* (3), *Providencia stuartii* (3), *Enterobacter cloacae* (1) and *Klebsiella pneumoniae* (1); for this group, antimicrobial resistance patterns included ampicillin/sulbactam for non-*Pseudomonas* spp. (100% of isolates), ciprofloxacin (76.9%), extended-spectrum cephalosporins (61,5%), gentamicin (61.5%), meropenem (11.5%) and piperacillin/tazobactam (11.5%). Co-resistance patterns included extended-spectrum penicillins/ciprofloxacin/gentamicin (38.4%), extended-spectrum penicillins/ciprofloxacin/extended-spectrum cephalosporins (30.8%), extended-spectrum penicillins/gentamicin/extended-spectrum cephalosporins (15.4%), extended-spectrum penicillins/meropenem/extended-spectrum cephalosporins (11.5%); one isolate was resistant to all tested antimicrobials.

Demographic and clinical characteristics between subjects who acquired MDRO or not were not significantly different, except that Alzheimer’s disease as the cause of dementia, was more frequent among MDRO acquirers (86.8 vs. 62.1%, *P* = 0.01). There were no significant differences between groups in exposure by antimicrobial class. Antimicrobial exposure by class was highly heterogenous, therefore we did not analyze clustering patterns of the samples according to the spectrum of action of each antimicrobial class.

**FIGURE 4:**
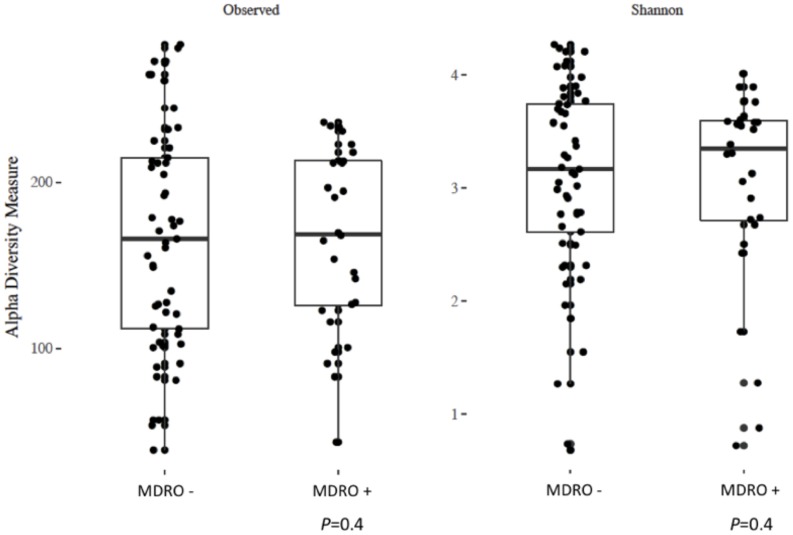
Analysis of alpha diversity based on observed number of ASV **(left panel)** and the Shannon index **(right panel)** between *a priori* fecal samples from study subjects who acquired MDRO (MDRO+) or not (MDRO–). Neither of the distributions were significantly different (*P* > 0.1) between the MDRO+ and MDRO- subjects. ASV, amplicon sequence variant; MDRO, multidrug-resistant organisms. Dots represent patient’s samples.

**FIGURE 5:**
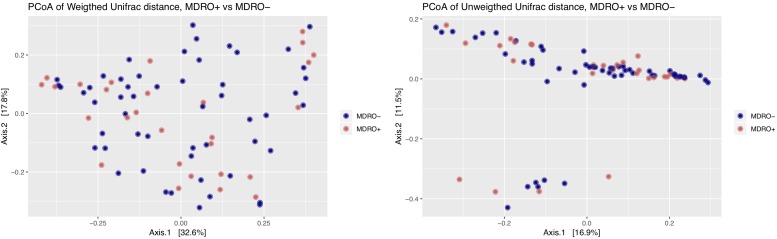
Principal coordinates analysis (PCoA) of weighted and unweighted UniFrac distances between study subjects according to MDRO acquisition status [MDRO + (orange); MDRO- (blue)]. Differences in clustering patterns were tested using the ADOINS test (*P* > 0.05). MDRO, multidrug-resistant organisms.

**FIGURE 6:**
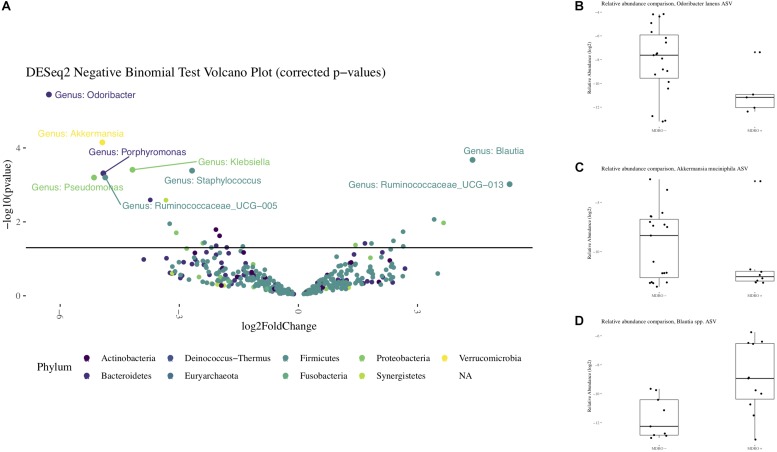
Differential representation of taxa from the 16S rRNA amplicon sequencing. Panel **(A)** Volcano plot with differentially abundant features observed between subjects who acquired MDRO or not (MDRO+ or MDRO-, respectively). Only features whose relative abundances remained significantly different after correcting for multiple comparisons are labeled (FDR < 5%). A negative log2 Fold Difference in relative abundance indicates the feature predominated among MDRO-, and vice versa for MDRO+. Panels **(B–D)**. Boxplots with the relative abundances of *Odoribacter laneus*, *Akkermansia muciniphila* and *Blautia* spp. among MDRO- and MDRO+ subjects. MDRO, multidrug-resistant organisms; FDR, false discovery rate.

Using 16S amplicon sequencing data, neither richness nor evenness, nor between-sample diversity analysis, performed by comparing UniFrac distances, differed significantly between subjects who acquired MDRO or not (Figures 4, 5). However, analysis of differentially abundant features between subjects who acquired MDRO or not identified several taxa that characterized each group. Among subjects who acquired MDRO, ASVs assigned to *Blautia* spp. [Log2 Fold-Difference (Log2 FD): 4.4, *Padj* = 0.03], and *Ruminococcaceae_UCG-013* (Log2 FD: 5.3, *Padj* = 0.04) were more abundant than on those who did not. Among subjects who did not acquire MDRO, ASVs corresponding to *Odoribacter laneus* (Log2 FD: 6.3, *Padj* = 0.002), *Akkermansia muciniphila* (Log2 FD: 4.9, *Padj* = 0.02), *Porphyromonas* spp. (Log2 FD: 4.9, *Padj* = 0.03), *Klebsiella* spp. (Log2 FD: 4.2, *Padj* = 0.03), *Staphylococcus* spp. (Log2 FD: 2.7, *Padj* = 0.03), *Pseudomonas* spp. (Log2 FD: 5.1, *Padj* = 0,03), and *Ruminococcaceae_UCG-005* (Log2 FD: 4.9, *Padj* = 0.03), predominated (Figure 6).

**FIGURE 7:**
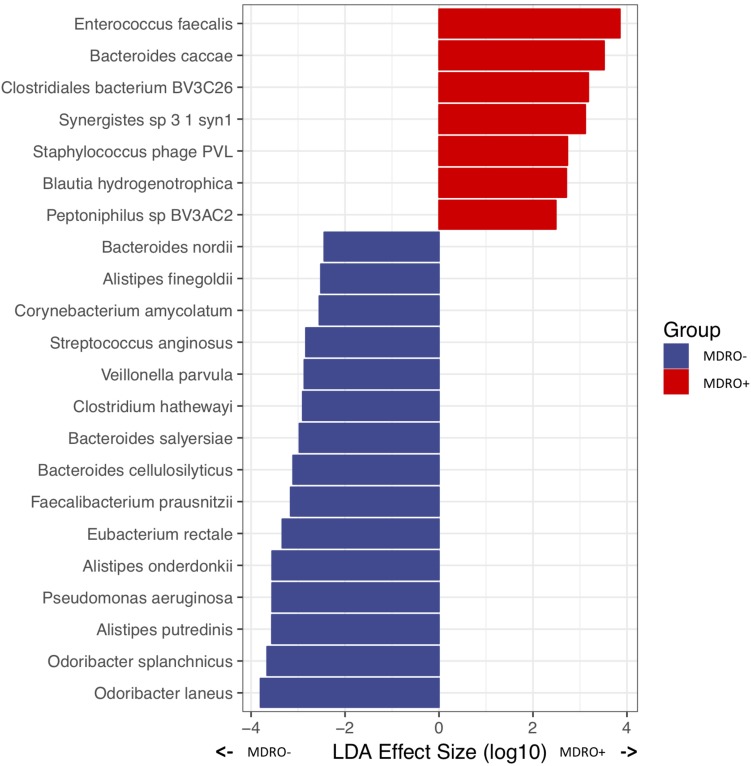
Differentially abundant microbial species in *a priori* samples from subjects who were MDRO- or MDRO+, as determined by whole metagenome shotgun (WMS) sequencing. MDRO, multidrug-resistant organisms.

Analysis of WMS sequencing showed that overall microbial diversity did not differ significantly between subjects who acquired MDRO or not. From a compositional perspective, Gammaproteobacteria were differentially more abundant among subjects who did not acquire an MDRO. At lower phylogenetic levels, several taxa characterized each group: within the Firmicutes, *Eubacterium rectale*, *Faecalibacterium prausnitzii*, *Clostridium hathewayi*, *Veillonella parvula* and *Streptococcus anginosus*, predominated in subjects who did not acquire MDRO, as did members of the Bacteroidetes, including *Odoribacter* spp. (*laneus* and splanchnicus), *Alistipes* spp. (*Putredinis*, *onderdonkii* and *finegoldi*), and *Bacteroides* spp. (*cellulosilyticus*, *salyersiae*, and *nordii*), as well as the Proteobacteria *P. aeruginosa*, and the Actinobacteria *Corynebacterium amycolatum*. Conversely, the relative abundances of Firmicutes such as *Enterococcus faecalis*, *Blautia hydrogenotrophica*, and *Peptoniphilus* spBV3AC2, as well as the Bacteroidetes *Bacteroides caccae*, and Synergistetes *Synergistes* sp. 3_1_syn1 were significantly higher among subjects who acquired MDRO (Figure 7). These findings show particular signatures in the composition of the fecal microbiome that can be consistently identified among subjects that acquire MDRO or not using different sequencing approaches.

### Analysis of the Fecal Resistomes With Respect to Acquiring MDRO

Finally, we asked whether or not the *a priori* abundance of AR was predictive of those who would acquire MDROs. In fact, we found that the median (IQR) absolute abundance of AR genes among subjects who did not acquire MDRO was significantly higher than in those acquiring MDRO [934 (625–1997) vs. 761 (540–932), respectively; *P* = 0.04]. The diversity of AR genes estimated by their within-sample richness was similar between the two groups. Beta diversity, as assessed by estimating the Bray–Curtis dissimilarity index between samples according to AR gene content did not show any specific clustering pattern according to MDRO acquisition or not. Although the rank order of AR gene superclasses within each group varied, no differentially abundant features regarding particular AR genes were observed between groups at the superclass level.

## Discussion

Using complementary approaches based on the analysis of 16S rRNA amplicon and WMS sequencing data (Schloss, 2018), we conducted a cross-sectional, metagenomic study to evaluate the fecal microbiome and its resistome among the elderly with advanced dementia, a population at high-risk for MDRO infections. We observed that the microbiome of this cohort was severely dysbiotic and, harbored a large number of diverse and unique AR genes compared to healthy controls. Moreover, we identified specific microbial signatures among subjects who acquired MDRO compared to those who did not acquire MDRO, suggesting that specific gut taxa may predispose to, or protect against, MDRO colonization.

The fecal microbiome of our study cohort was characterized by reduced microbial diversity and enrichment with pathogenic bacteria, suggesting that significant imbalances occur in the gut microbiome of elderly subjects exposed to antimicrobials. Since dysbiosis is known to increase with age and antimicrobial exposure (Biagi et al., 2010; Taur et al., 2012), it is not surprising that in our elderly population with universal antimicrobial exposure, fecal microbiome alterations were detected. Although Bacteroides and Firmicutes, the predominant phyla among healthy aged persons have been reported to represent >90% of the fecal microbiome (Biagi et al., 2010), these phyla represented only 62.4% in the group we studied. The lower percentage was due to the predominance of Proteobacteria, a well-described feature of the microbiome of hospitalized subjects exposed to antimicrobials (Araos et al., 2016), and in several disease states (Taur et al., 2012; Harris et al., 2016). High Proteobacteria abundance has been associated with an increased risk of subsequent infections caused by gram-negative bacteria (Taur et al., 2012). This Proteobacteria abundance may explain the predominance of gram-negative infections in the elderly population residing in nursing homes (O’Fallon et al., 2009).

The abundance of AR gene families and the number of different AR superclasses in the study cohort was four-fold and two-fold higher, respectively, than in the resistome of healthy younger persons. Clinically relevant AR genes also were very prevalent. Nearly all of the elderly subjects harbored AR genes for multidrug-efflux pumps and *cfr*, a highly mobile plasmid-mediated gene with the potential of conferring resistance to linezolid, was present in 52% compared to only 8.5% of healthy controls (Doern et al., 2016). Although the presence of the *cfr* gene may not have direct clinical relevance since it can be harbored by commensal microbiota, it still poses a risk via horizontal gene transfer to pathogenic bacteria. More than half of the AR gene families detected were identified only in the study cohort, including three-OXA-type Class D beta-lactamases which may confer resistance to narrow- and extended-spectrum cephalosporins, and carbapenems (Jeong et al., 2009), and may not be detected by standard phenotypic tests (Poirel et al., 2010; Antunes et al., 2014). Current methods for assessing the resistome may underestimate the contribution of the anaerobic commensal microbiota to the intestinal reservoir of AR genes, and the predominance of Proteobacteria in our cohort indicates a relationship of bacterial phylogeny with AR gene distribution consistent with previous data (Gibson et al., 2015; Feng et al., 2018). As such, we hypothesize that the enrichment of Gammaproteobacteria among subjects who did not acquire MDRO may explain the higher AR gene abundance in this group compared to those who acquired MDRO.

This large and diverse resistome, including genes associated with phenotypic resistance to antimicrobials of last resort, such as carbapenems and linezolid, demonstrates that the population of elderly persons, residing in long-term care facilities, serve as major reservoirs of AR genes. The predominant human gut resistome consists of chromosomally encoded AR determinants, with a relatively low capacity for dissemination (Ruppé et al., 2019). However, our finding of highly prevalent AR determinants associated with mobile genetic elements, such as *cfr* and *qnr* to a lesser extent, suggests that this population of elderly subjects with advanced dementia and frequent antimicrobial exposure represents a group where opportunities for horizontal gene transmission between commensal bacteria and human pathogens are increased (Penders et al., 2013). Ways to better steward antimicrobial use in this population become increasingly important (Boutrot et al., 2019).

Comparing differences between the microbiomes of subjects who acquired MDRO or not, we observed a significantly greater relative abundance of *A. muciniphila* among subjects in the latter group. These organisms have been considered biomarkers for a healthy intestine due to an inverse relation in abundance and intestinal disease, including Crohn’s and ulcerative colitis (Png et al., 2010). *A. muciniphila* also may have a protective role as efficient mucus layer colonizers outcompeting pathogen colonization (Belzer and de Vos, 2012). Thus, these bacteria may provide a protective role against MDRO gut colonization.

Based on both 16S rRNA and WMS sequencing data, we observed a significantly higher relative abundance of *B. hydrogenotrophica*, a human gut acetogen, among subjects who subsequently acquired MDRO. This finding contrasts with prior data suggesting that other *Blautia* species (in particular, *B. producta)* may contribute to human intestinal colonization resistance against VRE and *C. difficile* (Jenq et al., 2015; Caballero et al., 2017). More studies are needed to determine whether *B. hydrogenotrophica* may be useful as a microbial index of a dysbiotic microbiome, predicting MDRO acquisition. Further investigation is needed to clarify the potential role of other differentially abundant features, such as *O. laneus*, and their utility as biomarkers associated with the risk of MDRO acquisition.

Our study has several limitations. First, we performed a cross-sectional study nested in a longitudinal cohort. Therefore, the associations described between the distribution of exposures (microbiome features) and outcomes (MDRO acquisition) in our population cannot be considered as necessary for the causal chain. Nevertheless, our study describes a unique group of subjects at high-risk for MDRO acquisition and transmission with implications for improving infection control. Future studies should include additional control groups to better understand the role of age and antimicrobial exposure on the intestinal microbiome and resistome. Second, metagenomic prediction of AR genes has limitations inherent to the method, including falsely positive identifications, and lack of information at the functional level, as predictions may not translate into phenotypic antimicrobial resistance (Ruppé et al., 2019). However, we ameliorated these problems by using ShortBRED, a highly specific tool for predicting protein families including AR genes from WMS data (Kaminski et al., 2015). Third, microbiome assessment methods have several weaknesses that may increase the risk of misclassification bias. In particular, 16S rRNA amplicon sequencing is subject to primer and amplification bias which may lead to misrepresentation of the relative abundances of the community members. To overcome some of these limitations, we included a dual approach using 16S rRNA amplicon and WMS sequencing data (Munafò and Davey Smith, 2018; Schloss, 2018). Importantly, the assessments of the microbiome structure and composition were consistent between methods, but differences remained. As such, the variation observed in the analysis of differentially abundant features between subjects who acquired MDRO or not may have been determined by the different analytic approaches applied to each set of data (DESeq2 and LEfSe for 16S rRNA and WMS data, respectively).

## Conclusion

In conclusion, this study analyzed the fecal microbiome and its resistome in elderly subjects residing in nursing homes who were exposed to antimicrobials. Our results revealed that this patient population has a substantially dysbiotic microbiome and its resistome is composed of a large and diverse set of AR genes. The predominance of several taxa associated with acquiring MDRO or not suggests that there may be strategies targeting the microbiome which could prevent MDRO acquisition.

## Data Availability Statement

The datasets generated and analyzed for this study can be found in BioProject under the accession number PRJNA531921 (https://www.ncbi.nlm.nih.gov/bioproject/?term=PRJNA531921).

## Ethics Statement

The studies involving human participants were reviewed and approved by the institutional review board at Rhode Island Hospital, Providence, RI, United States approved this study. The patients/participants provided their written informed consent to participate in this study.

## Author Contributions

RA, MB, and ED’A conceived and designed the experiments. RA, TB, JU, and MR-H performed the experiments. RA, TB, JU, MB, and ED’A analyzed the data. RA and ED’A wrote the manuscript. RA, TB, JU, MR-H, MB, and ED’A edited the manuscript. All authors read and approved the final manuscript.

## Conflict of Interest

The authors declare that the research was conducted in the absence of any commercial or financial relationships that could be construed as a potential conflict of interest.
