# Supplementary material for: Fecal Microbiome Characteristics and the Resistome Associated With Acquisition of Multidrug-Resistant Organisms Among Elderly Subjects
Source: Front Microbiol. 2019 Sep 27;10:2260. doi: 10.3389/fmicb.2019.02260 (PMC6777474; doi:10.3389/fmicb.2019.02260)
Supplement: Supplementary file 1 [file Table_1.DOCX]

**Supplementary Table 1.** Complete list of antimicrobial resistance gene families predicted from WMS data, comparing the study population of elderly persons and healthy young subjects from the Human Microbiome Project (HMP).

| **Antimicrobial Resistance Gene (ARG) Families** | | |
| --- | --- | --- |
| **Unique to study elderly cohort** | **Shared between groups** | **Unique to the HMP young cohort** |
| AAA26613 | AAA22905 | AAM11666 |
| AAA26779 | AAB03644 | BAE96116 |
| AAA98484 | AAB51122 | CAM98046 |
| AAB47993 | AAC36915 | Chloramphenicol_Acetyltransferase_CAT_3 |
| AAC98092 | AAD26631 | NP_389192 |
| AAD12753 | AAD42180 | NP_840140 |
| AAD33243 | AAD42184 | YP_001187667 |
| AAD42181 | AAF71282 |  |
| AAD42182 | AAQ16274 |  |
| AAD42185 | AAZ98840 |  |
| AAD51059 | ABCAntibioticEffluxPump_10 |  |
| AAF01499 | ABF69686 |  |
| AAF24091 | ABG36114 |  |
| AAF72358 | ABN80187 |  |
| AAF72359 | ABO42050 |  |
| AAF72361 | ANT3_1 |  |
| AAF72362 | ANT6_0 |  |
| AAF72364 | ANT6_1 |  |
| AAK53556 | APH6_5 |  |
| AAK63223 | BAA07922 |  |
| AAN60217 | BAC77251 |  |
| AAQ16267 | baeR_2 |  |
| AAQ16268 | CAA26199 |  |
| AAQ16269 | CAC35724 |  |
| AAQ16271 | CAE48334 |  |
| AAQ16273 | CAL18627 |  |
| AAR96043 | CAM12479 |  |
| AAV74570 | CepA_3 |  |
| AAW30456 | Cfr23RibosomalRNAMethyltransferase_3 |  |
| AAY21388 | CfxA_11 |  |
| AAY52010 | emrB_10 |  |
| ABA71729 | FluoroquinoloneResistantDNATopoisomerase_2 |  |
| ABB99435 | FluoroquinoloneResistantDNATopoisomerase_28 |  |
| ABCAntibioticEffluxPump_0 | FluoroquinoloneResistantDNATopoisomerase_32 |  |
| ABP68837 | macA_3 |  |
| adeA-adeI_1 | macB_3 |  |
| adeB_1 | marA_1 |  |
| adeC-adeK-oprM_1 | MFSAntibioticEffluxPump_9 |  |
| adeC-adeK-oprM_5 | NP_268322 |  |
| adeR_0 | NP_344871 |  |
| adeS_1 | NP_414593 |  |
| BAA31456 | NP_414995 |  |
| BAA34300 | NP_416758 |  |
| BAA34540 | NP_417970 |  |
| BAB71966 | NP_418166 |  |
| BAC67147 | NP_418504 |  |
| baeR_0 | NP_418505 |  |
| baeS_1 | NP_478145 |  |
| blaI_0 | NP_752857 |  |
| blaR1_0 | NP_755678 |  |
| CAA28209 | NP_878013 |  |
| CAA36304 | NP_878017 |  |
| CAG27847 | P00384 |  |
| Chloramphenicol_Acetyltransferase_CAT_7 | P06571 |  |
| Chloramphenicol_Acetyltransferase_CAT_8 | P10337 |  |
| Chloramphenicol_Acetyltransferase_CAT_9 | P11504 |  |
| ClassC-AmpC_10 | P30898 |  |
| ClassC-AmpC_96 | P30899 |  |
| ClassD_33 | P51563 |  |
| ClassD_8 | P72533 |  |
| CTXM_83 | phoQ_1 |  |
| DHA_0 | RNDAntibioticEffluxPump_2 |  |
| EDK74221 | romA_0 |  |
| emrB_1 | soxR_5 |  |
| emrB_4 | SubclassB1_133 |  |
| emrB_6 | SubclassB1_149 |  |
| emrB_9 | SubclassB1_238 |  |
| emrE_0 | SubclassB1_257 |  |
| Erm23SRibosomalRNAMethyltransferase_7 | Tetracycline_Resistance_Ribosomal_Protection_Protein_9 |  |
| FluoroquinoloneResistantDNATopoisomerase_0 | WblE_0 |  |
| FluoroquinoloneResistantDNATopoisomerase_10 | WhiB2_1 |  |
| FluoroquinoloneResistantDNATopoisomerase_17 | YP_001005900 |  |
| FluoroquinoloneResistantDNATopoisomerase_18 | YP_001031495 |  |
| FluoroquinoloneResistantDNATopoisomerase_19 | YP_001096379 |  |
| FluoroquinoloneResistantDNATopoisomerase_3 | YP_001102018 |  |
| macA_1 | YP_001144149 |  |
| macB_2 | YP_001177494 |  |
| macB_4 | YP_001334578 |  |
| MacrolideGlycosyltransfer_0 | YP_001396002 |  |
| MFSAntibioticEffluxPump_12 | YP_001457897 |  |
| mprF_0 | YP_001480516 |  |
| mprF_1 | YP_001598107 |  |
| msbA_1 | YP_001764893 |  |
| msbA_3 | YP_001816601 |  |
| msbA_4 | YP_001834888 |  |
| msbA_5 | YP_001836040 |  |
| NP_040465 | YP_001965484 |  |
| NP_052694 | YP_001966009 |  |
| NP_052903 | YP_001969930 |  |
| NP_115321 | YP_002115369 |  |
| NP_249117 | YP_002239313 |  |
| NP_249118 | YP_002317674 |  |
| NP_249397 | YP_002382193 |  |
| NP_250709 | YP_002382998 |  |
| NP_251183 | YP_002384189 |  |
| NP_252799 | YP_002398143 |  |
| NP_252895 | YP_002559372 |  |
| NP_252896 | YP_002640141 |  |
| NP_252897 | YP_002773891 |  |
| NP_253065 | YP_002918846 |  |
| NP_253287 | YP_002929946 |  |
| NP_253288 | YP_002937728 |  |
| NP_370558 | YP_002939420 |  |
| NP_370559 | YP_187539 |  |
| NP_370576 | YP_195816 |  |
| NP_370577 | YP_310429 |  |
| NP_371207 | YP_403947 |  |
| NP_371219 | YP_405004 |  |
| NP_372857 | YP_410370 |  |
| NP_460867 | YP_542447 |  |
| NP_764021 | YP_603198 |  |
| NP_775043 | YP_672241 |  |
| NP_862236 | YP_691152 |  |
| NP_878015 |  |  |
| NP_878016 |  |  |
| NP_878023 |  |  |
| NP_931173 |  |  |
| NP_932197 |  |  |
| O87866 |  |  |
| P00484 |  |  |
| P07641 |  |  |
| P07944 |  |  |
| P13246 |  |  |
| P13249 |  |  |
| P14508 |  |  |
| P22616 |  |  |
| P26825 |  |  |
| P28585 |  |  |
| P30897 |  |  |
| P78218 |  |  |
| phoQ_0 |  |  |
| Q00014 |  |  |
| Q02736 |  |  |
| Q52424 |  |  |
| ramA_0 |  |  |
| RNDAntibioticEffluxPump_12 |  |  |
| RNDAntibioticEffluxPump_5 |  |  |
| robA_0 |  |  |
| soxR_0 |  |  |
| soxR_2 |  |  |
| soxR_4 |  |  |
| T44117 |  |  |
| TEM_137 |  |  |
| Tetracycline_Resistance_MFS_Efflux_Pump_19 |  |  |
| tolC_1 |  |  |
| tolC_3 |  |  |
| vanR_9 |  |  |
| YP_001038094 |  |  |
| YP_001096238 |  |  |
| YP_001101962 |  |  |
| YP_001140148 |  |  |
| YP_001345920 |  |  |
| YP_001348697 |  |  |
| YP_001420884 |  |  |
| YP_001420885 |  |  |
| YP_001451669 |  |  |
| YP_001468286 |  |  |
| YP_001668663 |  |  |
| YP_001713702 |  |  |
| YP_001713703 |  |  |
| YP_001798653 |  |  |
| YP_001844878 |  |  |
| YP_001967743 |  |  |
| YP_002258163 |  |  |
| YP_002269113 |  |  |
| YP_002333392 |  |  |
| YP_002633428 |  |  |
| YP_069175 |  |  |
| YP_185297 |  |  |
| YP_251227 |  |  |
| YP_254114 |  |  |
| YP_254125 |  |  |
| YP_271816 |  |  |
| YP_274443 |  |  |
| YP_298289 |  |  |
| YP_348389 |  |  |
| YP_349555 |  |  |
| YP_415591 |  |  |
| YP_473355 |  |  |
| YP_698526 |  |  |
| YP_790742 |  |  |
| YP_976075 |  |  |
